# Phase Behavior of Ionic Liquid-Based Aqueous Two-Phase Systems

**DOI:** 10.3390/ijms232012706

**Published:** 2022-10-21

**Authors:** Lirong Nie, Ziwei Zheng, Mingxia Lu, Shun Yao, Dong Guo

**Affiliations:** 1 School of Health Science and Engineering, University of Shanghai for Science and Technology, Shanghai 200093, China; 2 School of Chemical Engineering, Sichuan University, Chengdu 610065, China

**Keywords:** phase equilibrium, ionic liquid, aqueous two-phase system, salting-out agent, mechanism

## Abstract

As an environmentally friendly separation medium, the ionic liquid (IL)-based aqueous two-phase system (ATPS) is attracting long-term attention from a growing number of scientists and engineers. Phase equilibrium data of IL-based ATPSs are an important basis for the design and optimization of chemical reactions and separation processes involving ILs. This article provides the recent significant progress that has been made in the field and highlights the possible directions of future developments. The effects of each component (such as salting-out agents and ILs) on the phase behavior of IL-based ATPSs are summarized and discussed in detail. We mainly focus on the phase behavior of ATPSs by using ILs, expecting to provide meaningful and valuable information that may promote further research and application.

## 1. Introduction

As one kind of liquid-liquid extraction system, the aqueous two-phase system (ATPS) was originally discovered by Albertsson [1]. ATPS is composed of two immiscible liquid phases whose solvent in both phases is water. In its early stages, it consisted of two polymers, or polymers and inorganic salts. The two polymers, or a polymer and a salt, were incompatible at certain concentrations due to the repulsive interaction, thus forming two phases. In the ATPS, there is little difference in the properties of the two phases, which is beneficial for maintaining the activities and structures of the extracted substances. Most importantly, water acts as a major component in both phases, which provides a mild environment to effectively avoid the toxicity and environmental pollution caused by volatile organic solvents. As a whole, ATPS is considered as one of the excellent alternatives for traditional liquid-liquid extraction technology. Recently, it has widely been used in the separation and purification of biological macromolecules, metal ions, natural products, carbon nanomaterials, etc. [2,3,4,5,6]. Compared to the traditional liquid-liquid extraction method, ATPS is eliciting increased research interest due to its advantageous characteristics, such as being eco-friendly, having a short extraction time, low energy consumption, good biological compatibility, easy amplification and continuous operation [7,8,9,10]. However, the limited polarity interval of the coexistence of two phases has become the bottleneck that has been restricting its application.

Ionic liquids (ILs) are salts that, in contrast to common electrolytes, are liquid at low temperatures. Due to their ionic nature, ILs possess unique properties, such as negligible vapor pressure, non-flammability, thermal and chemical stability, tunable chemical structures and physical properties, and strong solubilization ability [11,12,13]. As a result of these excellent properties, ILs are often used as entrainers to form the binary or ternary liquid-vapor and liquid-liquid mixtures with various organic compounds, which are applied in the separation processes. Furthermore, the structural characteristics of ILs have an influence on the homogeneous and heterogeneous characteristics of mixtures [14,15]. Recently, with the continuous development of ILs in the field of extraction and separation, ILs are expected to be alternatives of volatile organic solvents to form ATPS. Generally, hydrophobic ILs are often used to form IL/water biphasic systems. However, given the high viscosity of the IL phase, possible denaturation would take place during extraction/separation of biomacromolecules when using simple IL/water biphasic systems. Thus, ILs are more compatible with the advantages of ATPS as an environmentally friendly extraction system. At the same time, ionic liquid-based aqueous two-phase system (IL-based ATPS) can effectively solve the problem of low extraction efficiency resulting from the unregulated polarity of traditional polymer-polymer or polymer-salt ATPS [16]. In 2003, Rogers and co-workers reported, for the first time, a ATPS constructed by hydrophilic IL (1-butyl-3-methylimidazolium chloride, [C_4_mim]Cl) and inorganic salt (K_3_PO_4_). Results indicated that the IL-based ATPS could overcome the limitations of the IL/water-extraction systems mentioned above [17]. Since then, significant progress has been made in the field of IL-based ATPSs [18,19,20]. The current research shows that ILs can form ATPS with salts [21,22], polymers [23,24,25,26] or surfactants [27,28]. Furthermore, the properties of ILs can be adjusted to expand the application range of the ATPS extraction system through the structure design in the anions and cations of ILs. The IL-based ATPS possesses shorter phase separation time and clearer two-phase interface than the ATPS composed of polymers. The application process of IL-based ATPS often involves many complex conditions. The stability evaluation of ILs under these conditions is particularly important. Moreover, ILs have high cost and environmental toxicity. The recovery of ILs after use is crucial for the development of green chemistry. However, unfortunately, few studies have reported on the above respects.

Although previous studies have revealed some important aspects of IL-based ATPSs, there are few systematic studies, especially concerning their properties, phase formation ability and phase equilibrium behavior. Moreover, the influence of components in IL-based ATPS on phase behavior are very important for the selection and design of extraction separation process. This review aims to provide a different perspective on IL-based ATPS from those recently published papers, majorly discussing the properties, the phase equilibrium and separation mechanisms. The discovery and development of ATPSs involving ILs are reviewed in detail. To sum up, the review mainly focuses on the following aspects: (1) the effects of the structures of ILs and salting-out agents on the properties of IL-based ATPSs; (2) the phase separation mechanism of IL-based ATPS; (3) the phase equilibrium behavior of various IL-based ATPSs. Furthermore, possible problems or challenges are also discussed, which may provide meaningful and valuable information to the relevant area and thus promote further research and application of IL-based ATPSs.

## 2. Properties of IL-Based ATPSs

**Figure 1:**
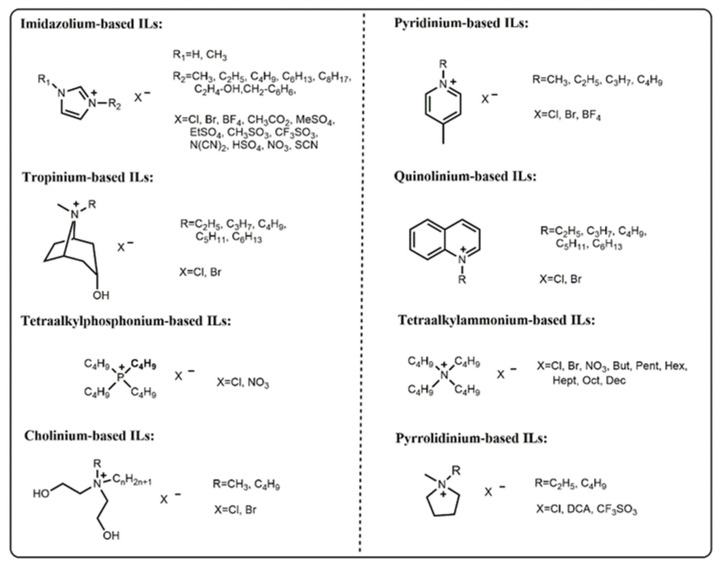
Chemical structure of the ILs commonly used for ATPSs.

The physical properties of the ATPSs at various concentrations and temperatures are indispensable requirements for the design and scaling up of separation process. The properties of IL-based ATPSs largely depend on the structure and property of ILs. Hydrophilic ILs have the advantages of many kinds of anions, low cost and hypotoxicity in comparison with hydrophobic ILs. Hydrophilic ILs are more widely used in the ATPSs. The chemical structure of ILs commonly used for ATPSs are listed in Figure 1. In the IL-based ATPSs, the chaotropic ILs act as the salting-in species and the anti-chaotropic ILs have the contrary role [29,30]. The use of ILs in ATPS can improve the phase polarities more adequately. These interesting and advantageous properties of the IL-based ATPS have motivated numerous studies of thermodynamic data, modeling, extraction and so on [31,32,33,34,35].

### 2.1. Effects of ILs Cations

**Figure 2:**
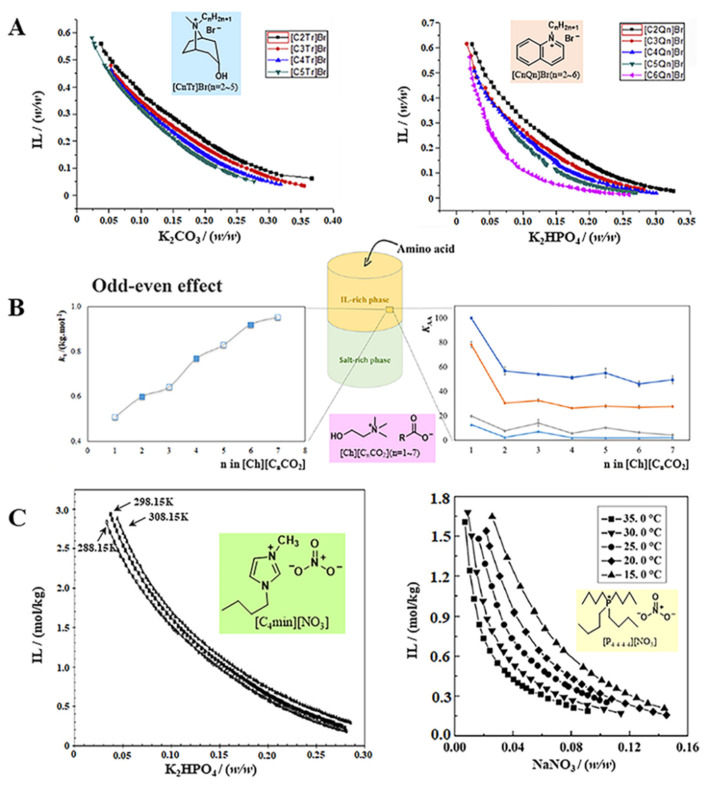
(**A**) Effect of ILs cations on the binodal curves [42]; (**B**) Odd-Even effect of the ATPS based on [Ch][CnCO_2_], comprising anions with odd and even alkyl chain lengths [43]; (**C**) Effect of temperature on the phase formation ability of different IL-based ATPS [44,45].

The cations of ILs usually consist of parent nucleus and side chains, which are easily modified. Most of the currently studied ILs are designed to have chaotropic cations that are salted-out by kosmotropic salts. For example, Bridges et al. [36] investigated phase behaviors of the IL-based ATPSs formed by [C_4_mim]Cl, 1-butyl-2,3-dimethylimidazolium chloride ([C_4_mmim]Cl), N-butylpyridinium chloride ([C_4_py]Cl), tetrabutylammonium chloride ([N_4 4 4 4_]Cl), and tetrabutylphosphonium chloride ([P_4 4 4 4_]Cl) with different inorganic salts, including K_3_PO_4_, K_2_HPO_4_, K_2_CO_3_, KOH, and (NH_4_)_2_SO_4_. The chaotropicity decreased in the order: [P_4 4 4 4_]Cl > [N_4 4 4 4_]Cl >> [C_4_py]Cl >> [C_4_mmim]Cl ≈ C_4_mim]Cl. Obviously, this order is due to the increased chaotropic nature of the salts resulting from the chemical differences in the cations. The two quaternary onium salts have highly shielded charge, which is located mostly on the heteroatom surrounded by four butyl chains. Therefore, they are easily salted out by salting agents. Compared to the quaternary onium salts, the charge of the pyridinium cation mostly located on the nitrogen is less shielded. The imidazolium-based ILs have charge diffused cations, leading to depressed melting points. The charge of imidazolium cation is evenly distributed on the two nitrogen atoms and the C2 carbon, which results in multiple interactions between the cation and the molecular water. Therefore, two imidazolium-based ILs are the most difficult ones to be salted out. The systems evaluated are made up of K_3_PO_4_ and imidazolium-based ILs, including [C_4_mim]Cl; [C_4_C_1_C_1_im]Cl or [C_6_mim]Cl [37]. Results showed that the phase separating ability of [C_4_mmim]Cl was between [C_4_mim]Cl and [C_6_mim]Cl. That is, the hydrogen bonds between the C2 hydrogen atom of imidazolium cation and water have less effect on the phase behavior. By using K_2_HPO_4_-KH_2_PO_4_ as the salting agents to control pH, Ventura et al. [38] compared the phase separation ability of four substituted alkyl ILs with the same chain length. The sequence for the phase formation ability for the various families is as follows: [C_4_C_1_pyr]Cl ≈ [C_4_mim]Cl < [C_4_C_1_pip]Cl < [C_4_C_1_py]Cl. According to the results, the ability of phase formation of above IL-based ATPSs was greatly affected by the IL cation molar volume. ILs cations with six-numbered heterocycles (such pyridiniun and piperidinium) have greater steric hindrance, and the hydrogen bond network of the surrounding water is more easily destroyed. Therefore, they are easier to be salted out than imidazolium- and pyrrolidinium-based ILs, with five-numbered heterocycles. Moreover, the density and viscosity measurements of both phases for ATPS based on phosphonium and imidazolium cations have also been reported. There are no significant differences in density values between IL-based ATPS and typical polymer-based systems. However, the phases containing phosphonium- or imidazolium-based ILs are far less viscous than the typical polymer-rich phases at the condition of close mass fraction compositions [39,40,41]. Additionally, the hydrophobic of ILs also affects the phase formation of IL-based ATPS. In general, the hydrophobic of ILs increases as the alkyl chain on cations lengthens, meaning stronger phase separation ability. This phenomenon was observed in the ATPSs based on two series of ILs, including n-alkyl-tropinium bromide ([C_n_Tr]Br, n = 2~5) and n-alkyl-quinolinium bromide ([C_n_Q_n_]Br, n = 2~6), were formed ATPS with different salts (Figure 2A) [42].

Many studies have found that when the carbon number of cationic side chain alkyl group in ILs was less than or equal to 6 (n ≤ 6), the ability of phase formation of IL-based ATPS was enhanced with the increase in the hydrophobicity of ILs [42,46,47,48]. However, an over high hydrophilic nature of the IL is not beneficial for the formation of ATPS. The appropriate increase in the length of non-polar alkyl chain can lead to a strengthened hydrophobic nature of ILs, resulting in a weaker affinity with water. Anomalous rules of the phase separation ability of ILs were observed when the carbon number of cationic side chain alkyl group is greater than or equal to 8 (n ≥ 8) [49,50]. This may be due to the self-aggregation structure formed by ILs in the system. In addition, the substitution of different functional groups on the side chain of ILs also affects the property of the phase forming system. For example, hydroxyl and allyl groups on side chain substituents on imidazolium cation could significantly reduce the phase separation ability of ILs. additionally, the effect of benzyl and n-heptyl groups on phase separation was not significant [47,51,52]. Compared to the benzyl and n-heptyl groups, the hydroxyl and allyl groups on side chain substituents exhibit the higher water affinity. The higher the affinity for water and/or hydrophilic nature of the IL, the less effective is the IL in promoting ATPS.

### 2.2. Effects of ILs Anions

Due to the variety of the structure of ILs anions, there is no unified standard for comparison. Therefore, the effects of ILs anions on the ability of phase formation have rarely been studied. In 2007, Pei and co-workers [49] compared, for the first time, the phase separation ability of the halogenide anions (Br^−^ and Cl^−^). When IL cations were 1-butyl-3-methylimidazolium ([C_4_mim]^+^) and 1-hexyl-3-methylimidazolium ([C_6_mim]^+^), the ability of phase formation of Br^−^ was obviously better than that of Cl¯. This can be explained by the different Gibbs energies of hydration (Δ*_hyd_G*) of Cl^−^ and Br- anions. The absolute value of Δ*_hyd_G* of Cl^−^ (−340 kJ/mol) is larger than that of Br^−^ (−315 kJ/mol). Therefore, the ILs with the Cl^−^ anion hydrate more water molecules than the ILs with the Br^−^ anion, resulting in difficulty in the phase formation as salt was added. Subsequently, a wide range of the ILs anions were studied, aiming at obtaining new insights regarding their ability toward the formation of IL-based ATPSs. On the basis of the IL cations, including 1-ethyl-3-methylimidazolium and 1-butyl-3-methylimidazolium, the influence of IL anions on the ATPS formation was assessed through their combination with chloride, bromide, acetate, hydrogensulfate, methanesulfonate, methylsulfate, ethylsulfate, trifluomethanesulfonate, trifluoroacetate, and dicyanamide. The results indicated that the ability of ILs for ATPS formation followed the order: [C_2_mim][CF_3_SO_3_] > [C_2_mim][C_2_SO_4_] > [C_2_mim][MeSO_4_] > [C_2_mim][Br] > [C_2_mim]Cl ≈ [C_2_mim][CH_3_CO_2_] > [C_2_mim][CH_3_SO_3_] and [C_4_mim][CF_3_SO_3_] > [C_4_mim][N(CN)_2_] > [C_4_mim][HSO_4_] > [C_4_mim][TFA] > [C_4_mim]Br > [C_4_mim]Cl ≈ [C_4_mim][CH_3_CO_2_] ≈ [C_4_mim][CH_3_SO_3_] [53]. With the increasing capacity of anions as hydrogen bond acceptor (i.e., hydrogen-bond alkalinity), the ability of ILs with the same cation for ATPS formation decreased [38]. This may be because the strong hydrogen-bond alkalinity of ILs is favorable to the interaction with water to form hydrate, which will lead to the weak phase separation ability. Moreover, most anions are small in size and do not have long hydrophobic alkyl chains. Therefore, the hydrogen bond interaction is considered to be the main factor affecting the phase separation process.

In some cases, the weak hydrogen-bond alkalinity of ILs can significantly affect the distribution of solute between two phases and reduce the extraction capacity of the ATPSs, which restricts to some extent the application of IL-based ATPS in the separation field [54,55]. Deive et al. [56] constructed the IL-based ATPSs consisting of high charge-density inorganic salts and several ethyl-methylimidazolium alkylsulfate ILs, [C_2_mim][C_n_SO_4_] (n = 2, 4, 6, or 8), at room temperature. The influence of different alkyl chain lengths in the anion on the formation of ATPS was investigated. The results indicated that the phase separation ability of ILs increased with the increase in the length of the alkyl chain in the IL-anion. Similarly, a set of ILs containing tetrabutylammonium cation and carboxylate anions were synthesized by Basaiahgari et al. [57] to form ATPS in presence of strong salting-out agent K_3_PO_4_. Furthermore, the influence of alkyl chain length of ILs’ anions on the phase splitting ability was evaluated. The resultant trend of phase formation among studied ILs was as follows: [TBA][But] < [TBA][Pent] ≈ [TBA]Br < [TBA][Hex] < [TBA][Hept] ≈ [TBA][Dec] < [TBA][Oct]. It can be assumed that the hydrophobicity of anions plays a dominant role in the phase formation. Jimenez et al. [58] used imidazolium-based ILs with different alkyl side chains and anions (chloride, bromide, acetate or dicyanamide) as phase-forming components of ATPS. It is observed that anions have a dramatic effect on ATPS immiscibility when a random co-polymer of ethylene oxide and propylene oxide monomers (namely, UCON) was used to form ATPS with different ILs. The area of the heterogeneous region follows the series OAc^−^ > Cl^−^ > Br^−^ > SCN^−^. At the same time, this order is consistent with the absolute value of Gibbs energy of hydration of these anions [59]. Moreover, amino acid ILs (AAILs) have the advantages of the tunable hydrophobicity and acid/base behavior, the low toxicity and biodegradability, which are widely used in ATPS [43,60,61,62,63]. Most recently, Korchak et al. [62] compared the phase separation ability of several AAILs with different amino acid anions, such as L-Leucine ([L-Leu]), L-Valine ([L-Val]), L-Lysine ([L-Lys]). Based on the lengths and the slopes of the tie lines, the ability of the studied systems (AAILs + inorganic salts) to phase separation increased in the following sequence: [Lys] > [Val] > [Leu] > Cl > Br. This result indicated that the heterogeneous region for amino acid ILs is wider than for halide ILs. However, an odd-even effect resulting from their structures was observed in the ATPS based on cholinium carboxylate ILs ([Ch][C_n_CO_2_] with n = 1~7, comprising anions with odd and even alkyl chain lengths) [43]. The Setschenow salting-out coefficients (*k*_s_) was determined to quantitatively describe the two-phase formation ability, which mainly depends on the properties of salts. The value of *k_s_* is proportional to the ion valence. The higher the *k_s_* value, the better salting out effect. As shown in Figure 2B, these ILs comprising even alkyl chains display slightly higher *k*_s_ values, meaning that they are more prone to being salted out or more easily phase separated. Moreover, the existence of an odd-even effect is also visible in the extraction performance of ATPS for four amino acids (L-tryptophan, L-phenylalanine, L-tyrosine, and L-3,4-dihydroxyphenylalanine). The result is the opposite. The ATPS formed by ILs with anions comprising odd alkyl chains lead to slightly higher partition coefficients of amino acids (*K_AA_*). The differences in partitioning could mainly arise from the dispersive interactions between these hydrophobic amino acids the IL anion aliphatic moieties.

To sum up, the overall phase separation ability and extraction efficiency of IL-based ATPS are governed by multiple factors prevailing at a microscopic level such as hydrophobicity, hydrogen bond accepting ability and dispersive interaction. The above research can provide new strategies for enhancing the phase separation ability of ILs.

### 2.3. Effects of Temperature

Temperature is a significant factor that affects the formation of the ATPSs. Generally, the heterogeneous region of the IL-based ATPSs varies to a certain degree as the temperature increases or decreases [18]. The influence of temperature on the properties of IL-based ATPS was assessed by several researchers. For instance, a recent review was reported by Chakraborty and co-workers [64]. Furthermore, the Merchuk equation and its fitting parameters were expressed as a function of temperature in the linear form with (T-T_0_) K as a variable. T_0_ was assumed as the reference temperature, 273.15 K [64,65,66,67]. The nonlinear expression of Merchuk equation is as follows:

(1) $$w_{1}=a\times exp\left(bw_{2}{}^{0.5}-cw_{2}{}^{3}\right)$$

where *w*_1_ and *w*_2_ represent the concentrations (in weight percent) of IL and salt, respectively. *a*, *b*, and *c* represent the fitting parameters.

It must be emphasized that the effect of temperature on ATPS is quite complex, varying differently for different systems. Based on the salting-out coefficient (*k*_s_) obtained from fitting the tie-line data, Zafarani-Moattar et al. explained the effect of temperature on the phase-forming ability of the IL [C_4_mim]Br + tri-potassium citrate system [65,66]. The results indicated that the value of *k_s_* increased with the decreasing of temperature. Higher phase-forming ability has a larger value of *k_s_*. Similarly, the ATPS composed of [C_4_mim][BF_4_] + (NH_4_)_2_SO_4_ + H_2_O at three different temperatures (298.15, 308.15, 318.15 K) was investigated by Wang et al. [67]. The solubility of IL decreases with the decrease in temperature. Therefore, the binodal curve shifts down, resulting in an expansion in the heterogeneous region. That is, a decrease in temperature leads to an increase in phase-forming ability. Novel ATPS composed of *N*-butylpyridinium tetrafluoroborate ([C_4_py][BF_4_]) and inorganic salts (Na_2_SO_4_ and (NH_4_)_2_SO_4_) were studied [68]. The two-phase region expanded with the decrease in temperature, which indicated that the low temperature was favorable for the phase separation. Moreover, the reliability of tie-lines was evaluated by Othmer-Tobias and Bancroft equations. The tie lines at low temperatures possessed longer TLL, which mean better phase separation ability at low temperatures. Therefore, the studied ATPS is beneficial for the extraction of targets that are inactivated at higher temperature, such as proteins. This trend has also been observed for the ATPSs constituted by ILs and carbohydrates, such as [C_4_mim][BF_4_] and glucose [69], fructose [70], sucrose [71,72] or maltose [73]. The strong interaction between the IL and carbohydrates is not conducive to the formation of ATPS. This may be because low temperature can destroy the interaction between the IL and carbohydrates, leading to expansion of the biphasic region. Malekghasemi et al. [44] studied the effect of temperature on the phase formation ability of the IL [C_4_mim][NO_3_] + K_2_HPO_4_ + water ATPS. As shown in Figure 2C, the decrease in the temperature slightly caused the expansion of the two-phase area. Although the above phenomenon is different from the traditional polymer + salt system [74,75,76,77], it can be found in most ILs + salt ATPSs. However, there are a few ATPSs composed of IL and inorganic salt, which undergo the expansion of immiscibility regime with the increase in temperature [45,78]. This trend mainly occurs in some ILs containing tetrabutylphosphonate cations. For example, a hydrophilic IL tetrabutylphosphonate nitrate ([P_4 4 4 4_][NO_3_]) was used for the formation of an ATPS with NaNO_3_. It is clear from Figure 2C that the phase separation ability of IL was significantly enhanced with an increase in temperature, suggesting that the lower concentration of [P_4 4 4 4_][NO_3_] and NaNO_3_ was required for the formation of ATPSs as the temperature increased [45].

Additionally, the different trends to induce the liquid-liquid demixing at different temperatures were observed for the IL-based ATPS containing polymers, such as polyethylene glycol (PEG) and polypropylene glycol (PPG) [79,80,81,82]. The solubility of PEG and PPG in water mainly depends on the mutual PEG/PPG-water hydrogen bond interactions. In conventional ATPS composed of PEGs and ILs, the temperature affects the liquid-liquid demixing process by disturbing the hydrogen-bonding interactions. Moreover, there are lower critical solution temperatures (LCST) and upper critical solution temperatures (UCST); it refers to two different stimuli-responsive behaviors of the system at different temperatures. For the LCST system, the phase separation occurs when the temperature drops to a certain level. Whereas the UCST system shows the opposite thermal response behavior, that is, phase separation occurs when the temperature rises to a certain degree. Therefore, some ILs-PEG/PPG ATPSs have phase behaviors in response to external thermal stimuli. At a low IL concentration, the system is the type of LCST. Additionally, the system transforms into UCST type at high IL concentration [83]. For example, aqueous solutions of PEG polymers with different molecular weights (600, 1000, 2000, and 3400 g mol^−1^) and several protic ILs were mixed and their ability to form ATPS at several temperatures was assessed [84]. An increase in the immiscibility region or the phase-forming ability with the increase in temperature for ammonium acetate ([NH_4_][OAc]), propylammonium acetate ([C_3_NH_3_][OAc]) and butylammonium acetate ([C_4_NH_3_][OAc])-based ATPSs was observed. This belongs to the typical LCST. The high temperature can decrease the mutual IL–PEG/PPG hydrogen-bonding interactions, which facilitates the creation of ATPS. However, an increase in the temperature reduced the biphasic region of the hexylammonium acetate ([C_6_NH_3_][OAc]) and butylammonium butanoate ([C_4_NH_3_][But])-based ATPSs. In brief, the temperature influence on the phase separating ability is a quite complex phenomenon. Multiple factors contribute to the result, such as hydrogen-bonding interaction, concentration of ILs and the types of coexisting phase forming components.

## 3. Mechanism of Phase Separation

The phase separation mechanism of the ATPS composed of ILs and salts has been well established. The salting-out effect is the major force affecting the phase separation and the extraction [85]. In general, ILs are in a stable dispersed state in water. However, the abilities of salts and ILs to adsorb water molecules are different. When an aqueous solution containing salt is added to the IL aqueous solution, the formation of IL-based ATPS is a process of the competition between IL and salt for water molecules. On the one hand, the hydrophilic and hydrophobic properties of ILs affect their phase forming ability. For example, Ren et al. used five ILs to construct ATPS with K_3_PO_4_, K_3_C_6_H_5_O_7_, and K_2_CO_3_ [86]. The results showed that phase forming ability of ILs increased with the increase in their hydrophobicity. That is, the hydrophobicity of ILs is beneficial to the formation of ATPS [3,87,88]. On the other hand, the property of salts has an influence on the phase formation process. When the added salts contain kosmotropic ions (such as CH_3_COO^−^, SO_4_^2−^, HPO_4_^2−^, Mg^2+^, Ca^2+^, Li^+^, H^+^, OH^−^, etc.), water molecules around kosmotropic ions have a more regular arrangement order and lower free energy due to the polarization of kosmotropic ions. The tendency of two-phase separation is consistent with the ability of salt ions to form hydration complexes. Moreover, the stronger the hydration ability of salt ions, the easier it is to repel IL and form the second phase. The salting-out ability can be related to the Gibbs free energy (Δ*_hyd_G*) and the entropy (Δ*_hyd_S*) of hydration of the salt ions. The salting-out strength of the kosmotropic salts follows the Hofmeister series [89,90,91,92]. Compared to the chaotropic ions, the kosmotropic ions have large, negative Δ*_hyd_G* values, due to the formation of the structured water around them. Therefore, it is easier to form the ATPS by adding kosmotropic salts rather than chaotropic salts. As the kosmotropic ions and ILs move closer to each other, H_2_O molecules near ILs are gradually taken away by kosmotropic ions, resulting in the decrease in solubility of ILs. Then, the phase separation of IL-based ATPS is completed.

Compared to the IL-based ATPS formed with salts, the mechanism of IL-polymer ATPS is more complex. Although many studies have confirmed that the salting-out effect is the main force for IL-based ATPS with PEG/PPG [93,94,95,96,97], the phase separation principle of some ATPSs constructed by ILs and polymers does not completely follow the salting-out phenomenon. For instance, the phase separation mechanism of ATPS based on imidazolium-based ILs and PEG was investigated by Freire and co-workers [30]. The IL or polymer can form independent solvent effect with H_2_O molecules, caused by hydrogen bonds between water and IL or polymer. Additionally, the solvation determines the phase separation mechanism of this system. Similarly, Mourao et al. [98] also constructed the ATPS by mixing cholinium-based ILs and PEG with different molecular weight, respectively. In this system, the interaction between IL and PEG-600 is stronger than that between PEG-600 or IL and water. Thus, the phase separation mechanism is mainly determined by the interaction between PEG-600 and IL. The solvation of ILs in PEG aqueous solutions is very complex and the solvation process depends on the balance of all possible interactions (PEG-water, PEG-ILs, water-ILs) [84,99].

Additionally, the H-bond alkalinity and aggregation structures of ILs regulated by their components and functional groups play an important role for forming ATPS. Here, the aggregation structures often refers to the nature of poly(ionic liquid)s. Poly(ionic liquid)s are a type of ionic polymer obtained by polymerization of IL monomers, or grafting IL units onto the polymer backbone. Generally, the functional groups (such as double bond) on the structure of IL monomers are required to form poly(ionic liquid) by the polymerization. The hydrogen-bond interaction between ILs and water will be enhanced as the increase in hydrogen-bond alkalinity, which is not conducive to phase separation. Furthermore, the aggregation structures of ILs can change the interaction between ILs and water molecules, thus affecting the phase separation rule of ATPS [100]. The physicochemical property of poly(ionic liquid) can be tailored by changing the characteristic functional group of IL monomers. The hydrophobic interaction is the main driving force for micelle formation of ILs in water. When the IL monomer contains hydrophobic groups, the hydrophobicity of poly(ionic liquid) increases, resulting in an increase in its aggregation and phase separation ability.

## 4. Phase Equilibrium of the IL-Based ATPSs

**Figure 3:**
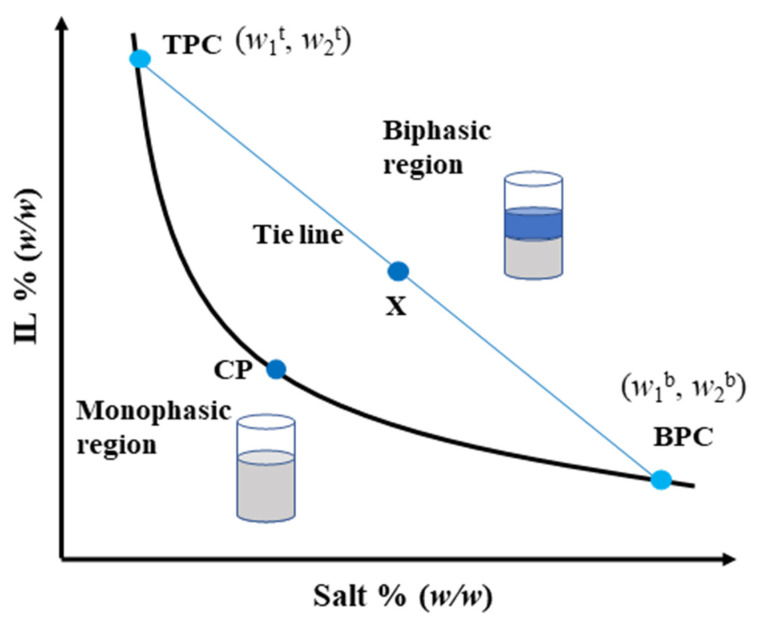
Conceptual diagram of an ATPS constituted of IL and salt, where the ordinate axis represents the IL concentration in % (*w*/*w*) and the abscissa axis the salt concentration in % (*w*/*w*).

The IL-based ATPS is formed by two phases, both consisting mainly of water. These phases are composed by water and IL or a salting-out agent. Under specific thermodynamic conditions, the IL-based ATPS typically has a top phase (TP) rich in IL (extract phase) and a bottom phase (BP) rich in salting-out agent. As shown in Figure 3, the formation of the ATPS is based on the composition of the two-phase system through a rectangular phase diagram that expresses the TP and BP concentrations in mass percentage % (*w*/*w*) [101]. The binodal curve refers to the line to separate the homogeneous from the heterogeneous regions. The upper side of the binodal curve is a two-phase system, and the lower side is a single-phase system. Under the same conditions, the larger the area of the biphasic region is, the stronger the phase separation ability is. For an ATPS prepared at the composition X, TPC and BPC reflect the top phase composition and the bottom phase composition, respectively. CP indicates the critical point. In addition, the tie lines (TL) are also obtained from the phase diagram. These lines are straight lines that connect global mixing points to their corresponding phase compositions (TPC and BPC). As the length of TL decreases in the diagram, the compositions become closer and closer. This behavior continues until the compositions become equal in the CP and the system is monophasic [102].

The phase diagram of the ATPS is instructive for its application in the extraction. It is of great significance to understand the genesis and influencing factors of IL-based ATPS for its innovative development. There are many factors affecting the phase diagram of IL-based ATPS, which not only depend on the structure and properties of ILs, but also are restricted by other coexisting phase forming components, such as salts, polymers, surfactants, amino acids and saccharides. The following categories of these influencing additives are discussed.

### 4.1. Salts

**Figure 4:**
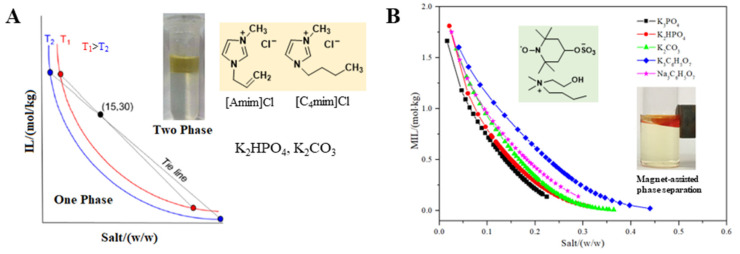
Effect of different salts on phase equilibrium behavior of imidazolium-based IL ATPS (**A**) and cholinium-based MIL ATPS (**B**) [103].

Within the scope of this work, salts are the most commonly used second phases formers besides ILs. As one of the major components of ATPSs, the effect of inorganic salts on the phase equilibrium was investigated by many researchers. It was reported that IL-based ATPSs could be formed by adding appropriate amounts of inorganic and organic salts (such as K_2_HPO_4_, K_3_PO_4_, K_2_CO_3_, KOH, NaOH, Na_2_HPO_4_, (NH_4_)_2_SO_4_, KCl, NaCl, K_3_C_6_H_5_O_7_ and Na_3_C_6_H_5_O_7_) into the aqueous solution of IL. In the research of Tanimura et al., two hydrophilic ILs, including 1-allyl-3-methylimidazolium chloride ([Amim]Cl) and [C_4_mim]Cl, were used to from the ATPS by adding the inorganic salts (K_2_CO_3_, K_2_HPO_4_) (Figure 4A). The phase separation mainly depends on the salting-out effect of inorganic salt in the bottom phase. In the same composition, the [C_4_mim]Cl/K_2_HPO_4_ system is more likely to undergo a two-phase separation at lower temperature conditions, suggesting that the phase-separation ability of the inorganic salt K_2_HPO_4_ is stronger than that of K_2_CO_3_ [103]. As a good indicator of the molecular partitioning behaviors in conventional ATPSs, the hydrophobicity index (HF) between the top and bottom phases was characterized by the distribution coefficients (*K*_aa_), of amino acids. Results showed that the HF value of this IL-based ATPS was almost consistent with an ATPS composed of PEG and salt. Recently, as a new class of the ILs, choline amino acid ILs ([Ch][AA]) were explored so as to construct the ATPS with different salts including (NH_4_)_2_SO_4_, KH_2_PO_4_, Na_2_CO_3_, and K_3_PO_4_ [104]. Compared to traditional ILs, [Ch][AA] are less toxic, more biodegradable, and have good air and water stability. Thus, the ATPS composed of [Ch][AA] and salt are often applied to the extraction and separation of food samples as an environmentally friendly extraction method [105].

More significantly, as one of functionalized ILs, magnetic ionic liquid (MIL) demonstrates a response to an external magnetic field. Imidazolium-based MIL [C_4_mim][FeCl_4_] was firstly synthesized by Hayashi et al. [106] in 2004. Since then, MILs have become a hotspot of research in field of extraction and separation [107,108,109,110,111]. Recently, remarkable progress has been made in the field of MILs and the related ATPS are stimulating interest [112,113,114,115]. Yao et al. prepared three MILs based on the tetramethylguanidinium ([C_n_TMG][TEMPO-OSO_3_], n = 2,3,4) to form ATPS with different inorganic salts. The authors found that (NH_4_)_2_SO_4_, Na_2_SO_4_, KOH, NaOH, NaCl as well as KCl could not drive any MIL aqueous solution to separate into two phases. Therefore, K_2_HPO_4_, K_2_CO_3_ and Na_2_CO_3_ were used as the salting-out agents for this ATPS. Among them, both K_2_HPO_4_ and K_2_CO_3_ have wide range of biphasic regions, indicating that they are better phase forming salts in comparison with Na_2_CO_3_ [114]. Most recently, a MIL-based ATPS was investigated by our group [115]. In our study, five cholinium-based MILs with the piperidinyloxy radical anion were synthesized for the first time and formed ATPS with inorganic or organic salts. For the five investigated MILs, the abilities of salts for phase-separation are in the following order: K_3_PO_4_ > K_2_HPO_4_ > K_2_CO_3_ > Na_3_C_6_H_5_O_7_ > K_3_C_6_H_5_O_7_. The salting-out ability of the anions follows the order: PO_4_^3−^ > HPO_4_^2−^ > CO_3_^2−^ > C_6_H_5_O_7_^3−^ when they share the same cation. Additionally, the salting-out ability of cations follows the order: Na^+^ > K^+^ for the citrate-based salts. The results are in accordance with the order of the absolute values of Δ*_hyd_G* and Δ*_hyd_S* for these ions [32]. That is, the increasing entropy may be the driving force for the two-phase formation [116]. Moreover, it is clear from Figure 4B that the MIL-rich phase (top phase) can be easily attracted by a magnet. Under an external magnetic field, the phase assembly and separation become more time-saving and easier, which make these systems superior to the common IL-based ATPS.

Although ILs and salts are present in the system as ions, the two phases of the ATPS remain electrically neutral, which is confirmed by Bridges et al. [36]. Similar to the polymer-salt ATPS, the salting-out ability of kosmotropic salt is consistent with the Hofineister series. Thus, it is directly related to their Δ*_hyd_G*. Most ILs as chaotropic salts are easily salted out by kosmotropic salts. In the IL-salt ATPSs, the main force of phase formation is salting-out effect, resulting from the formation of water-ion complexes and the increase in cavity surface tension [117,118]. Compared to the water molecules, the kosmotropic ions (e.g., HPO_4_^2−^, SO_4_^2−^, OH-, CO_3_^2−^ and PO_4_^3−^) exhibit the stronger interaction with water molecules, which are beneficial to the formation ATPS. Conversely, chaotropic ions (e.g., Cl^−^, NH_4_^−^, K^+^, and H_2_PO_4_^−^) find difficulty in forming ATPSs with ILs because the interactions between these anions and water molecules are weaker than those between water molecules. Finally, some of the salts commonly used in the IL-based ATPSs are listed in Table 1.

### 4.2. Polymers

Compared to common IL-based ATPS, the polymer-IL-based ATPS is more special. In the IL-based ATPS containing polymer, the neutral molecules (such as PEG and PPG) are the most commonly used polymers. Moreover, ILs are electrically charged salts. Therefore, ILs are the true sense of the salting-out agents in this ATPS, which is similar to the conventional polymer-salt ATPS. In 2007, Visak et al. reported the phase behavior of imidazolium-based ILs ([C_n_mim]Cl, n = 2~10) and PEG-3500 (*M*_w_ = 3500 g·mol^−1^) in water for the first time [131]. The ILs with long side chains in cation ([C_n_mim]Cl, n = 6, 8, 10) can promote the hydrogen bond interaction between PEG and water, resulting in the solubilization effect for PEG. At the same time, the presence of long alkyl chains makes the IL form micelles and other self-aggregation structures, which also acts as a cosolvent effect. ILs with short side chains ([C_n_mim]Cl, n = 2, 4) also could solubilize PEG at low concentrations, but could precipitate PEG at high concentrations. This may be due to the fact that ILs are both hydrophobic and hydrophilic, which are caused by the non-polar alkyl chain and the polar part of the anion or cation, respectively. Subsequently, the phase diagrams of various ILs and PEGs with different molecular weights in water were determined by Freire et al. [30]. The ability of phase formation was closely related to the hydrophobicity of PEG. The phase separation ability was enhanced with the increase in the molecular weight of PEG as a consequence of the increased hydrophobic properties of PEG. In the study of Tomé et al. [132], the experimental results combined with molecular dynamics (MD) simulations and density functional theory (DFT) calculations were provided to understand the molecular-level mechanisms behind the formation of ATPS composed of ILs and polymers. As a proof of principle, the experimental ternary phase diagrams composed of an IL ([C_4_mim]Cl), PEG-1500 (*M*_w_ = 1500 g·mol^−1^) and water were determined at two distinct temperatures. The results showed that this ternary IL-PEG-water system was of Type “0”. That is, the binary pairs (such as IL-PEG, IL-water and PEG-water) are completely miscible yet are able to form two-phase systems at given compositions of the ternary mixtures. Distinct from what happens in IL-salt-based ATPS, the formation of IL-PEG-based ATPS was controlled by the IL anion solvation by water, which resulted in the destruction of the hydrogen bond interaction between the IL anion and the hydroxyl groups of the polymer. When water is introduced into the PEG-IL binary system, the hydrogen bonds formed by IL and PEG are replaced by the stronger water-IL anion hydrogen bonds. Therefore, this mechanism of the ATPS formation is here labelled as a “washing-out” phenomenon, given the analogy with the washing process. In brief, it has herein been shown for the first time that IL-polymer-based ATPS was a result of a ‘‘washing-out’’ phenomenon, and not of a salting-out effect of the IL over the polymer as assumed in the past few years.

**Figure 5:**
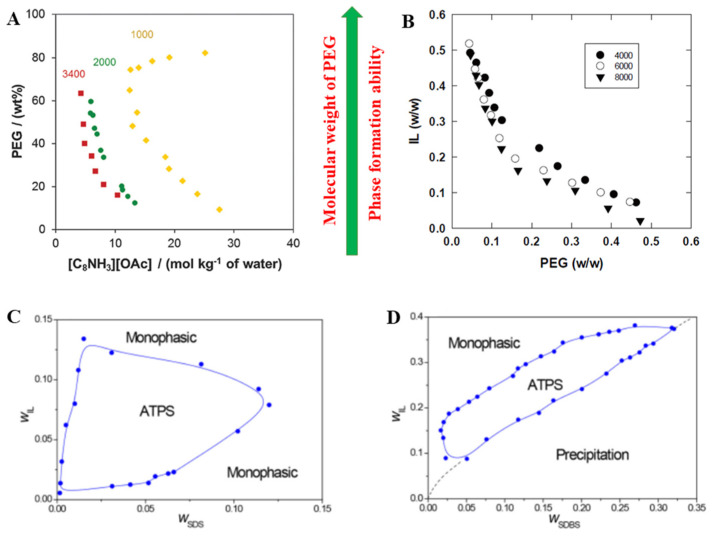
Effect of PEG molecular weight on phase formation ability of IL-based ATPS composed of [C_8_NH_3_][OAc] + water + PEG (**A**) and [C_6_mim][C_12_SO_3_] + water+ PEG (**B**) [84,133]; Phase diagrams (25 °C) of ternary systems of [C_2_pi][BF_4_] + SDS + H_2_O (**C**) and [Phpi][BF_4_] + SDBS + H_2_O (**D**) [134].

A recent extensive study on the formation of aqueous biphasic systems (ABS) using aqueous solutions of protic ILs and PEG was performed by Cláudio and co-workers [84]. A series of different molecular weight PEGs (600, 1000, 2000, 3400 g·mol^−1^) were selected to study the effect of the molecular weight of PEG for the formation of IL-based ATPS. Among them, the PEG-600 was unable to induce ATPS formation when mixed with the IL octylammonium acetate ([C_8_NH_3_][OAc]). For other polymers, the ability of the polymer to induce liquid-liquid demixing follows the order: PEG-3400 > PEG-2000 > PEG-1000 (Figure 5A). The results obtained indicated that polymers with the higher molecular weight were more able to promote phase separation. Ola et al. employed an ATPS formed by the IL 1-hexyl-3-methyl imidazolium dodecyl sulfonate ([C_6_mim][C_12_SO_3_]) and PEGs with a higher molecular weight [133]. The same trend was presented for the above ATPS. It is well-known that the closer a curve is to the origin of the coordinates, the lower the polymer concentration required for the formation of two phases and the stronger is the phase-forming ability of the polymer. As shown in Figure 5B, the phase formation ability was in the following order: PEG-8000 > PEG-6000 > PEG-4000. That is, the larger the molecular weight of PEG, the smaller the concentration of both PEG and IL necessary to form a two-phase system. The tendencies observed might be related to two factors: (i) the increase in the molecular weight increased the hydrophobicity of the phases, leading to consequent reduction in its water solubility/affinity; (ii) the PEG-IL miscibility increased by increasing the number of terminal –OH groups per PEG molecule.

Alongside PEG, PPG is also one of the commonly used polymers. Different from PEG-IL-based ATPS, PPG is more hydrophobic. So, the increase in hydrophobicity of cationic parent core and side chain of IL can enhance the interaction between IL and PPG, which is not conducive to phase separation [135,136]. Liu et al. employed two PPGs with distinct molar mass and poly(ethylene glycol)-block-poly(propylene glycol)-poly(ethylene glycol) (EO_10_PO_90_) to form environmentally friendly ATPS with four cholinium-based ILs, such as cholinium glycollate, cholinium propionate, cholinium lactate and choline chloride [81]. The results showed that the phase-forming ability of the polymers in cholinium IL-based ATPSs follows the order: EO_10_PO_90_ > PPG-1000 > PPG-400. Obviously, the order of their hydrophobic ability and molar mass is in agreement with the order of their phase-forming ability. Such a phenomenon is also observed for the PEG + IL ATPS. In the research of Neves et al. [79], a vast number of imidazolium-based ILs and PPG-400 were formed ATPS. Then, ^1^H NMR (Nuclear Magnetic Resonance) spectroscopy and COSMO-RS (Conductor-like Screening Model for Real Solvents) were used to obtain the molecular-level mechanisms which rule the phase splitting. For some systems, the IL-PPG-400 pairs were completely miscible, revealed to be of type “0”. All evidence suggested that the formation of PPG-IL-based ATPS was controlled by the interactions established between the IL and PPG. For a given IL concentration, we can move from the monophasic-biphasic-monophasic regimes only by increasing the amount of PPG in the system, or vice-versa. Therefore, these phase diagrams can be recognized as interesting separation approaches. In addition, the rise in temperature was beneficial to the formation of ATPS, which was consistent with the LCST phase behavior. Of course, the cholinium-based ILs with low toxicity and high biodegradability can also form ATPS with suitable polymers, such as polyethylene glycol dimethyl ether 250 (PEGDME-250) or PPG-400 [24,137]. It can be summarized as follows: among all PPG polymers, the PPG-400 are the most commonly used in IL-based ATPS. Since it is thermoresponsive, PPG-400 can be recovered from aqueous solution simply by heating the solution above the LCST.

Among all the IL-based ATPS studied hitherto, the more recent polymer-IL-based ATPS boost their applicability since they increase the hydrophilic and hydrophobic range of the coexisting phases, thus allowing for more selective separations to be achieved. Compared to other ATPS, the polymer-IL-based ATPS showed a different partitioning behavior. Moreover, both species (polymers and ILs) are able to act as salting-out agents. This property opens possibilities for the design of an appropriate and selective ATPS [138,139]. Some polymers used in the IL-based ATPSs are summarized in Table 2.

### 4.3. Surfactants

For the ATPS composed of surfactants and ILs, there was very limited research until 2011. In 2011, Wei et al. reported for the first time that the IL [C_4_mim][BF_4_] and a surfactant sodium dodecyl benzene sulfonate (C_18_H_29_SO_3_Na, SDBS) were mixed in water at a certain concentration to form the ATPS [143]. The borderlines of the different specific regions in the phase diagrams were determined using the turbidity titration method. In their work, the formation of SDBS-IL-based ATPS may be due to the formation of very large size micelle aggregates. The authors confirmed the result by Transmission Electron Microscopy (TEM) and Steady-state Fluorescence Quenching Measurements (SFQM) methods. The average size of the micelle aggregates in the upper phase and bottom phase was much larger than that in water. The larger micelles possessing smaller density aggregate in the upper phase, while the relatively small micelles remain in the bottom phase. Therefore, the bottom phase was slightly smaller than the upper phase in the micelle size. Moreover, this phase separation phenomenon was found to be likely due to the existence of micelle aggregates with quite a large size. The phenyl group attached to the -SO_3_^−^ in SDBS may conduct cation-π interaction with the cation ([C_4_mim]^+^) of [C_4_mim][BF_4_]. This interaction makes the butyl side chain align along the alkyl chain of the surfactant in the palisade layer of the micelle surface close to the phenyl moieties, which increases the size of micelle aggregates. In order to verify this mechanism, the author also studied the phase forming ability of different surfactants and found that only the surfactant containing a benzene ring can form a two-phase system with IL. Subsequently, the above ATPS was successfully used to extract the Sudan dyes from food samples [144]. Furthermore, inorganic salts played pivotal role on forming the ATPS because of intermicellar and intramicellar interaction. Thus, the inorganic salt (NH_4_)_2_SO_4_ was selected to maintain the stability of ATPS. This was mainly because the interaction between water molecule and SO_4_^2−^ was stronger than that between water molecules. Similarly, the ATPS formed by the IL [C_6_mim]Cl and SDS was reported for the determination of sulfonamides in blood [145]. In their work, four kinds of salts including K_2_CO_3_, (NH_4_)_2_SO_4_, K_2_HPO_4_ and NaCl were chosen to investigate the effect of ionic strength on the ATPS. Among them, Cl^−^ as chaotropic ions, the interaction with water molecules was relatively weak and solubilizing effect may be produced. With HPO_4_^2−^ as the kosmotropic ion, the interaction with water molecules was stronger than the interaction between water molecules, thereby facilitating the formation of ATPS [17,146]. Therefore, K_2_HPO_4_ was added to this system.

More recently, Lu et al. [134] constructed two kinds of ATPSs formed by 1-ethylpiperazinium tertrafluoroborate ([C_2_pi][BF_4_])/sodium dodecyl sulfonate (SDS) and 1-phenylpiperazinium tertrafluoroborate ([Phpi][BF_4_])/SDBS. At room temperature, their phase diagrams of two systems were shown in Figure 5C,D. Moreover, the water solubility of two anionic surfactants with the solution maintaining clear and transparent was determined by mass fraction. Although the water solubility of SDBS is lower than that of SDS, the addition of IL effectively increases the solubility of SDBS compared with that of SDS, while the experimental temperature is below the Krafft point of neat SDBS (38 °C) but higher than that of neat SDS (9 °C). Additionally, the critical micelle concentration (CMC) value of surfactants was also measured, and the CMC value for neat SDBS (1.2 × 10^−3^ mol/L) is slightly lower than that of SDS (8.2 × 10^−3^ mol/L). The lower the CMC is, the lower the concentration required for micelle formation is. The addition of [C_2_pi][BF_4_] into aqueous solution of SDS can significantly reduce the CMC of SDS. The cation ([C_2_pi]^+^) with positive charge is able to influence the electric double layer of the micelles formed by anionic surfactant, reducing the minimum concentration for micelle formation. During the micelle formation, the [C_2_pi]^+^ may participate in the self-assembly of micelles as cosurfactant, which is distributed in the interfacial layer mainly through electrostatic interactions with the negatively charged headgroup of surfactant. In a word, the packing effect induced by the re-assembly of micelles formed by IL cation and surfactant anions is a critical factor. That is, the presence of [C_2_pi][BF_4_] can improve the surfactant efficiency by reaching the minimum surface tension at a lower SDS concentration. However, as shown in Figure 5D, an insufficient amount addition of [Phpi][BF_4_] into the aqueous solution of SDBS leads to precipitation.

Furthermore, nonionic surfactants, such as octylphenol polyethoxylene (Triton X and Tween family, were also considered to be able to form ATPS with ILs. In H_2_O-IL-Triton X ternary system, H_2_O-IL, H_2_O-Triton X, and IL-Triton X can be completely miscible under room temperature conditions. However, an immiscibility window in the ternary region is observed, whose area is enlarged with the increase in temperature (known as island-type ternary system) [147,148]. For example, the detailed phase diagram of this island-type ternary system ([C_2_mim][C_2_SO_4_]+Triton X+water) has been published by Alvarez et al. [147]. The immiscibility window occurred only in the ternary region, while binary mixtures involved in the system were completely miscible. Moreover, the area of the immiscibility window increased obviously with the rising of temperature. This group further studied the phase separation of [Ch]Cl-Triton X-water ternary system. Compared with the IL [C_2_mim][C_2_SO_4_], the hydrophilic IL [Ch]Cl and nonionic surfactant leaded to a relatively larger two-phase region [148]. It is called IL-nonionic surfactant ATPS, in which the two-phase system consists of an IL-rich phase and a nonionic surfactant-rich phase [149]. More importantly, anionic species exhibit a high partitioning coefficient between the IL-rich phase and the surfactant-rich phase, which may be useful for stripping the anionic species off nonionic surfactant aqueous solution. As well as this, the novel IL-nonionic surfactant ATPS was formed in the [Ch]Cl-Tween 80-water ternary system [150]. In IL-surfactant-water ATPS, the IL was often used as salting out agent. The ability of the IL to salt out aqueous solutions of polymers such as PEG-600, organic compounds such as tetrahydrofuran or nonionic surfactant such as Tween 20 and Tween 80, has been researched and published [82,151,152]. Recently, the ATPS for the ternary mixtures (Tween 20 or Tween 80 + [N_1 1 1 2OH_][C_4_H_5_O_6_] + H_2_O) were constructed to evaluate the segregation capacity of IL [152]. In terms of the IL chemical properties, the lower hydrophilicity prefers to a reduced capacity to establish hydrogen bonds with water molecules existing in the nonionic surfactant aqueous solutions, resulting in a smaller biphasic region, as can be checked from the experimental data previously reported [150,153]. Moreover, the role of the surfactant mainly depends on its hydrophobicity. It is expected that a surfactant with lower hydrophilicity is more favorable to the formation of two phases. This analysis can also be quantitatively performed by checking the HLB, which is a dimensionless parameter going from 0 to 20 (from high to low hydrophobicity). Hence, the area of the biphasic regions follows the order: Tween 80 (HLB = 15.0) > Tween 20 (HLB = 16.7). Apart from that, the interest of the present IL can be revealed by the comparison of the size of the immiscibility region with those provided by conventional salts such as K_3_PO_4_ or K_3_C_6_H_5_O_7_ [154,155]. Finally, some surfactants recently used in the IL-based ATPSs were summarized in Table 3.

### 4.4. Amino Acids

In addition to inorganic salts with a high charge density, some small molecular organic compounds with a low charge density can also be used as the salting agents to build the ATPS with ILs, such as amino acid organic compounds. In 2007, Zhang et al. [158] reported for the first time that a hydrophilic IL ([C_4_mim][BF_4_]) could form ATPS with three amino acids (including glycine, L-serine, and L-proline), respectively. Different from the ATPS composed of K_3_PO_4_ and [C_4_mim]Cl [17], the upper phase is the glycine-rich phase, and the lower phase is the [C_4_mim][BF_4_]-rich phase. By forming ATPS with the IL at different temperatures (298.15K, 308.15K and 318.15K), it was found that the ATPS was more easily formed at lower temperatures. This may be due to the decreased miscibility between the [C_4_mim][BF_4_]-rich phase and the amino acid-rich phase with the increasing of temperature. Moreover, the ATPSs of three amino acids at 298.15 K were compared in their study. As a result, their tendency to form an ATPS with the IL [C_4_mim][BF_4_] is in the order of glycine > L-serine > L-proline. What’s more, the authors investigated another two hydrophilic ILs ([C_2_mim][BF_4_] and [C_4_mim]Cl) to form ATPS with the above three amino acids. Unfortunately, however, they failed to form an ATPS with these amino acids no matter how the ratio between the IL and amino acids was adjusted. Due to the stronger hydration ability of the above two hydrophilic ILs, amino acids seemed to salt out from the transparent phase when both ILs were added. Therefore, not all hydrophilic ILs can form ATPS with amino acids. On the basis of the above research, Domínguez-Pérez et al. [159] selected the ILs with the same cation 1-butyl-3-methylimidazolium ([C_4_mim]^+^) to form ATPS with different amino acids, such as L-lysine, D, L-lysine HCl and L-proline. The IL anion influence on ATPS formation was assessed through its combination with [BF_4_]^−^, [CF_3_SO_3_]^−^, and [N(CN_2_)]^−^ anions. Results indicated that the phase formation ability of three ILs was in the order: [C_4_mim][BF_4_] > [C_4_mim][CF_3_SO_3_] > [C_4_mim][N(CN)_2_]. The above sequence was also observed for IL-based ATPS formed by the addition of K_3_PO_4_ [53]. Thus, the amino acids under study behave similarly to salting-out inducing salts. The phase separation mechanism of IL-based ATPS formed by the addition of amino acids can be explained by the competition between the amino acids and the IL ions for the creation of water-ion hydration complexes. This is closely related to the anion’s hydrogen-bond basicity of the ILs. The hydrogen-bond basicity (β) data of three [C_4_mim]-based ILs were reported by Lungwitz et al. [160]. The smaller hydrogen-bond basicity value of IL is, the stronger its phase forming ability is. As the value of β increases, the hydrogen-bond accepting strength of the IL anion increases, which enhances its ability to be preferentially hydrated, resulting in a lower ATPS formation capacity of the IL. Additionally, the phase formation ability of amino acids to form ATPS follows the order: L-lysine ≈ D, L-lysine HCl > L-proline. It is not difficult to find that the more water-soluble amino acid is, the easier it is to form hydrate with IL, and thus the stronger the ability to separate out IL.

Compared with inorganic salts, amino acids have weak salting-out ability as salting-out agents. They promote phase separation by forming a water-amino acid complex to build an ATPS. Amino acids reduce the ionic strength of the solution, which can weaken or prevent the ion exchange between the two phases. This not only contributes to the practical application of IL-based ATPS, but also enables more efficient and convenient recycling of IL. Further insight into the salting-out inducing mechanism of amino acids can be obtained in the previous research [159,161]. According to the effects of a series of amino acids on the mutual solubilities of water and imidazolium-based IL, the salting-in and salting-out phenomena are the result of a delicate balance among water-amino acid side chain, IL-amino acid side chain and water-IL interactions, which are determined by the relative affinities of the biomolecule side chains to water and to IL. That is to say, the strength of the phase separation ability of amino acids greatly depends on their hydrophilicity. The stronger the hydrophilicity of an amino acid is, the higher its solubility in water is, and the stronger its ability to form hydrate is, which can then precipitate IL from aqueous solution. Moreover, the IL-amino acid ATPS has the same temperature sensitivity as IL-inorganic salt ATPS. Generally, the immiscibility region formed by IL and amino acid decreases with the increase in temperature, meaning that other phase forming component need to be added to facilitate phase separation. Therefore, the lower temperature is favorable for the formation of ATPS containing IL and amino acid. At present, only imidazolium-based ILs have been found to form ATPS with amino acids, which need further study.

### 4.5. Saccharides

Low molecular weight saccharides with specific structures also can be mixed with IL in water at a certain concentration and then phase separation can occur to form an ATPS. Similarly, the hydrophilic IL ([C_4_mim][BF_4_]) was firstly used to form ATPS with the fructose by Zhang and co-workers [70]. It was found that the ATPS can be formed over a wide component range and the effect of the temperature on the phase equilibria is obvious within the fructose concentration changing from 3 to 40%. Unfortunately, what they reported is just focused on the [C_4_mim][BF_4_]. The experiment was relatively simple and unrepresentative. Later, Chen et al. [69,73] systemically investigated the phase behaviors of 1-alkyl-3-methylimidazolium derivatives [C_n_mim]X (n = 2 to 10, X = Cl^−^, Br^−^, BF_4_^−^)-carbohydrate-H_2_O system. The results found that [C_n_mim]Cl (n = 2 to 10) and [C_n_mim]Br (n = 2 to 10) aqueous solutions never formed ATPSs with carbohydrates (such as glucose, sucrose, maltose, and xylose) in a wide temperature range (242.15K to 373.15K). However, carbohydrates can induce phase separation of not only the reported hydrophilic IL ([C_4_mim][BF_4_]), but also another homologous hydrophilic IL ([C_3_mim][BF_4_]) aqueous solution in the investigated conditions. The effect of carbon number of alkyl chain on imidazolium ring on phase separation ability was also studied. The two-phase area of [C_4_mim][BF_4_] system was larger than that of [C_3_mim][BF_4_] system. For hydrophobic ILs ([C_n_mim][BF_4_], n = 5 to 10), the addition of carbohydrate can reduce their mutual solubility with water, and therefore promote the formation of two-phase system. Moreover, the more glucose is added, the lower mutual solubility of IL and water is. By comparing the slope of tie-line (STL), the phase separation ability of the investigated carbohydrates followed the order: glucose > maltose > sucrose > xylose. More importantly, it is also found that ILs-rich phase and glucose-rich phase can be reversed by adjusting the amounts of glucose.

Obviously, all of the above-mentioned ILs are fluorinated-based ILs, which have emerged recently due to their remarkable performances, namely in the recovery of contaminants either in gaseous mixtures or in liquid effluents [162]. In addition to the commonly used imidazolium-based ILs, other fluorinated-based ILs are also used to form the ATPSs with different carbohydrates. In the research of Ferreira et al. [157], the novel ATPSs were developed by mixing a series of perfluoromethanesulfonate- and perfluorobutanesulfonate-based ILs and a large number of carbohydrates (monosaccharides, disaccharides and polyols) aiming at establishing more benign alternatives to the salts commonly used. In order to gain a better insight into ATPS formation involving ILs and carbohydrates, different ILs and carbohydrates were used to evaluate the influence of the structures of both components on the phase formation. The influence of the IL anion, in particular the size of the fluorinated alkyl chain length, was investigated by using the ILs comprising the same cation. Compared to anion [CF_3_SO_3_], the IL anion [C_4_F_9_SO_3_] behaved as the stronger two-phase promoter, indicating that the size of the fluoroalkyl chain of the anion has an effect on the formation of two-phase systems with carbohydrates. The longer fluorinated alkyl chain of the anion renders it more hydrophobic, and diminishes its affinity towards water, thus making it easier to separate out of the aqueous media. Then, three kinds of IL cations, including [N_1 1 1 2OH_]^+^, [C_2_C_1_py]^+^ and [C_2_mim]^+^, were selected to understand the role of the cation core in the process of phase splitting. Due to the higher hydrophilicity of cholinium-based ILs [163], it is not easy to induce phase separation in presence of a carbohydrate aqueous solution. In previous studies, cholinium-based ILs usually only form ATPS with strong salting-out salts, such as K_3_PO_4_ [163] or polymers [82]. The ability to form two phase systems is higher for [C_2_C_1_py]^+^ than for [C_2_mim]^+^. IL cations containing 6-carbon rings (such as pyridinium and piperidinium) formed ATPS more easily than those cations containing 5-carbon rings (such as imidazolium and pyrrolidinium). The above results suggested that the steric hindrance of the cation is also suggested to play important roles in water/IL interactions in addition to the hydrophobicity of ILs.

Nevertheless, the ability of carbohydrates to induce liquid-liquid demixing is mainly related to their characteristics and particularly to its hydration extension, which is determined by the interactions between carbohydrate and water, especially hydrogen-bonding. All the structures of carbohydrates have diverse –OH groups with dual donor-acceptor character and can establish hydrogen bonds with water and act as salting-out/sugaring-out species. In general, the more hydroxyl groups there are in a carbohydrate structure, the easier it is to form the hydration complexes by hydrogen bonding with water molecules, the stronger its phase separation ability is [164]. In this line, arabinose bearing 4 hydroxyl groups is expected to be a weaker two-phase promoter than all the other monosaccharides investigated with 5 hydroxyl groups. Likewise, noticeably different phase formation ability of polyols was observed following the order: maltitol > D-sorbitol > xylitol. The above trend is exactly consistent with the order of the number of hydroxyl groups they have. Maltitol is the largest polyol with more –OH groups, followed by D-sorbitol that has one more –OH group than xylitol. These experimental results also confirmed this expectation. Specially, for the disaccharides, including maltose and sucrose, despite they have the same number of –OH groups, maltose exhibits a higher ability to salt-out the IL when compared to sucrose. It could be because the six-membered pyranose rings in maltose makes it more likely to interact with water than the five-membered furanose ring in sucrose. Finally, some saccharides commonly used in the IL-based ATPSs were listed in Table 3.

Based on some relevant research, some major insights into the phase formation mechanism can be derived. The ability of ILs and saccharides to promote phase separation in aqueous media mainly depends on their hydration ability. Essentially, the phase separation is the process in which ILs and saccharides compete for the water molecules. When the saccharide aqueous solution is added to homogeneous IL solution, the two solutes compete for the water molecules. There is a “migration” of solvent molecules away from the ions of the IL towards the carbohydrate, which result in the decreasing of the solubility of the ionic solute in water. The water molecules may be structured around the saccharide and the hydrogen-bond interaction between saccharide and water is reinforced. That is, the sugar molecules are likely to win the competition since they have the innate higher affinity for water molecules than the IL and, hence, can establish stronger interactions with the solvent. On basis of their structures, the interaction of first-shell water molecules with solute primarily is with the hydroxyl groups of saccharides. When the saccharide concentration reaches a certain level, saccharide and water dissociate because of the interactions between saccharide and saccharide [165]. On the other hand, H_2_O-mediated clusters is formed by the attraction between the ions of IL as the concentration of IL increases [166]. The phase separation occurs when the cluster size is sufficiently large. In brief, two differently structured microphases of water are formed with the addition of saccharide to homogeneous IL solution. As the amount of IL or saccharide or in the solution increases, the stability of this microemulsion is disrupted, leading to the coagulation of the droplets (turbidity) and occurrence of phase separation. The competition of IL and saccharide for water molecules can cause the dehydration of IL ions. In this case, the saccharides are usually employed as salting-out agents [167].

## 5. Conclusions

ATPS formulation combines two chemicals whose aqueous solutions are immiscible under certain conditions (composition and temperature). The traditional ATPS is a liquid-liquid two-phase system which is formed by two polymers or polymer and salt in water. Recently, the rise of ILs provides a new direction for the exploration of new ATPS forming agents. As a structurally designable solvent, ILs can be adapted to different ATPSs by tailoring their structures. Taking into account the promising results reported in the literature reviewed, IL-based ATPS has many advantages shared by ILs (e.g., non-volatility, non-flammability, high thermal stability, structural designability and no need to use volatile organic solvent) and ATPSs (e.g., simple, high-efficiency, quick phase separation and gentle biocompatible environment). It is an excellent alternative to the traditional liquid-liquid two-phase system with competitive advantages at present when environmental problems are paid more and more attention.

Although ILs have good effects and industrial prospects in the ATPS extraction techniques, there are still some challenges associated with the IL-based ATPS. For example, the high viscosity and high costs as well as the recovery of the ILs after extraction process could limit their industrial application. Generally, high temperature can reduce the viscosity of ILs but may affect the property of the ATPS. It is also unsuitable for the extraction of heat-sensitive substances. Furthermore, more attention should be paid to the economics of recycling and reusing ILs. On the one hand, it is important to control the fabricating cost and energy consumption throughout the process, such as reducing the raw material cost and optimizing the process route. On the other hand, recycling ILs after use is another cost-saving approach. What’s more, the following knowledge is urgently needed before practical application of the IL-based ATPSs: (1) the basic physicochemical data, microstructure and properties for the IL-based ATPSs; (2) the phase separation mechanism; (3) the rules for the ILs selection for specific analytes in IL-based ATPSs.

The research results so far may expand the application of ILs, and more importantly, the work may provide new separation systems which may be potentially applied in biological, pharmaceuticals, and environmental engineering. As a kind of acknowledged extraction separation medium, ILs are actually not totally environmentally friendly and pollution-free. Moreover, ILs are difficult to be biodegraded due to their stable chemical properties. Current trends in IL-based ATPS gear toward employing more environmentally friendly approaches to comply with green analytical chemistry requirements. Therefore, there is a lot of hard work to be carried out on the development of IL-based ATPSs. It is expected that an environmentally benign and potentially ATPS will grow out of this review in the near future.

## Figures and Tables

**Table 1:** Some salts commonly used in the IL-based ATPSs.

Salts	ILs	Temperature/K	Refs.
K_3_PO_4_, K_2_HPO_4_, K_2_CO_3_, KOH, and (NH_4_)_2_SO_4_	[C_4_mim]Cl, [C_4_mmim]Cl, [C_4_py]Cl, [N_4 4 4 4_]Cl, [P_4 4 4 4_]Cl	298.15	[36]
K_2_HPO_4_, KH_2_PO_4_	[C_4_C_1_pyr]Cl, [C_4_mim]Cl, [C_4_C_1_pip]Cl, [C_4_C_1_py]Cl	298.15	[38]
K_3_PO_4_, K_2_CO_3_, K_2_HPO_4_, K_3_C_6_H_5_O_7_, Na_3_C_6_H_5_O_7_, NaH_2_PO_4_	[C_2_Tr]Br, [C_3_Tr]Br, [C_4_Tr]Br, [C_5_Tr]Br, [C_2_Qn]Br, [C_2_Qn]Br, [C_3_Qn]Br, [C4Qn]Br, [C_5_Qn]Br, [C_6_Qn]Br,	298.15~318.15	[42]
K_3_PO_4_, K_2_CO_3_, Na_2_CO_3_, (NH_4_)_2_SO_4_	[C_2_mim][C_n_SO_4_] (n = 2, 4, 6, or 8)	298.15	[56]
K_3_PO_4_, K_2_CO_3_	[Cnmim]X (n = 4, 8; X = [Lys], [Val], [Leu], Cl, Br)	298.15	[62]
K_2_CO_3_, Na_2_HPO_4_, Na_2_SO_4_, Na_3_C_6_H_5_O_7_, Na_2_CO_3_, K_2_HPO_4_, KH_2_PO_4_, (NH_4_)_2_SO_4_, (NH_4_)_3_PO_4_, K_3_PO_4_, NaNO_3_	[Ch][L-Pro], [Ch][L-Cys], [Ch][L-His], [Ch][L-Val], [Ch][L-Ser], [Ch][L-Met], [Ch][L-Ala]	298.15	[63]
K_2_HPO_4_	[Ch][C_n_CO_2_] (n = 1~7), [Ch]Cl	298.15	[43,119,120]
K_2_CO_3_	[C_4_mim][NO_3_], [C_6_mim][NO_3_], [C_2_mim]OAc	288.15~308.15	[76,121]
NaNO_3_, NH_3_NO_3_	[P_4 4 4 4_][NO_3_], [N_4444_][NO_3_]	288.15~308.15	[45]
K_3_PO_4_, K_3_C_6_H_5_O_7_, K_2_CO_3_	[C_4_MDEA]Br, [C_6_MDEA]Br, [C_8_MDEA]Br, [C_10_MDEA]Br, [C_4_BDEA]Br	298.15	[86]
K_2_CO_3_, K_2_HPO_4_	[Amim]Cl, [C_4_mmim]Cl	278.15~318.15	[103]
(NH_4_)_2_SO_4_, KH_2_PO_4_, Na_2_CO_3_, K_3_PO_4_	[Ch][Ala], [Ch][Gly]; [Ch][Lys], [Ch][Arg]	298.15~338.15	[104]
K_2_HPO_4_, K_2_CO_3_, Na_2_CO_3_	[C_2_TMG][TEMPO-OSO_3_], [C_3_TMG][TEMPO-OSO_3_], [C_4_TMG][TEMPO-OSO_3_]	298.15	[114]
K_3_PO_4_, K_2_HPO_4_, K_2_CO_3_, K_3_C_6_H_5_O_7_, Na_3_C_6_H_5_O_7_	[N_1 1 2 2OH_][TEMPO-OSO_3_], [N_1 1 3 2OH_][TEMPO-OSO_3_], [N_1 1 4 2OH_][TEMPO-OSO_3_], [N_1 1 5 2OH_][TEMPO-OSO_3_]	298.15~318.15	[115]
Na_3_PO_4_, (NH_4_)_3_PO_4_	[C_4_mim]BF_4_	288.15~318.15	[122]
NaNO_3_	[N_4441_][NO_3_]	298.15	[123]
(NH_4_)_2_SO_4_, NaH_2_PO_4_, Na_2_SO_4_, Na_2_HPO_4_, K_2_HPO_4_, NaCl, Na_3_C_6_H_5_O_7_	[C_4_mim][CF_3_SO_3_], [C_4_mim]Cl, [C_4_mim][BF_4_], [C_2_mim]Br, [C_4_mim]Br, [C_6_mim]Br, [C_8_mim]Br	278.15~318.15	[124]
Na_3_PO_4_, Na_2_CO_3_, Na_2_SO_4_, Na_3_C_6_H_5_O_7_, K_3_C_6_H_5_O_7_, NaH_2_PO_4_, NaCl, MgCl_2_, CaCl_2_	[C_4_mim][CF_3_SO_3_]	278.15~318.15	[125]
K_3_C_6_H_5_O_7_, (NH_4_)_3_C_6_H_5_O_7_, K_2_C_4_H_4_O_6_	[EOMiM]Br	288.15~308.15	[126]
(NH_4_)_3_C_6_H_5_O_7_, Na_2_C_4_H_4_O_4_	[C_4_py]OTF	298.15~328.15	[127]
Na_3_C_6_H_5_O_7_	[C_2_mim]DCA, [C_3_mim]DCA, [C_4_mim]DCA, [C_6_mim]DCA, [C_4_C_1_pyr]DCA	298.15	[128]
K_3_PO_4_	[C_4_mim]Cl, [C_4_mim]Cl; [C_4_C_1_C_1_im]Cl, [C_6_mim]Cl, [C_2_mim][CF_3_SO_3_], [C_2_mim][C_2_SO_4_], [C_2_mim][MeSO_4_], [C_2_mim][Br], [C_2_mim]Cl, [C_2_mim][CH_3_CO_2_], [C_2_mim][CH_3_SO_3_], [C_4_mim][CF_3_SO_3_], [C_4_mim][N(CN)_2_], [C_4_mim][HSO_4_], [C_4_mim][TFA], [C_4_mim]Br, [C_4_mim]Cl, [C_4_mim][CH_3_CO_2_], [C_4_mim][CH_3_SO_3_], [TBA][But], [TBA][Pent], [TBA]Br, [TBA][Hex], [TBA][Hept], [TBA][Dec], [TBA][Oct], [Ch][Leu], [Ch][Ala], [Ch][Gly], [Ch][Lys]	298.15	[17,37,53,57,129]
K_3_PO_4_, K_2_HPO_4_	[Ch][Pro], [Ch][Cys], [Ch][Ala], [Ch][His], [Ch][Met]	298.15	[130]

**Table 2:** Some polymers commonly used in the IL-based ATPSs.

Polymers	ILs	Temperature/K	Ref.
PEGDME-250, PPG-400	Ch[L-Ala]	298.15~318.15	[24]
UCON	[C_2_mim]Cl, [C_2_mim]Br, [C_2_mim][SCN], [C_2_mim][OAc]	288.15~308.15	[58]
PPG-400	[N_4444_]Cl, [P_4 4 4 4_]Cl, [C_4_mim][C_2_H_5_SO_4_], [C_4_mim][CF_3_SO_3_], [C_4_mim][N(CN)_2_], [C_4_mim][SCN], [C_5_mim]Cl, [C_6_mim]Cl, [C_7_mim]Cl	298.15~318.15	[79]
PPG-400, PPG-700, PPG-1000	DIMCARB, DPCARB, DACARB, DBCARB	288.15~308.15	[80]
EO_10_PO_90_, PPG-1000, PPG-400	Cholinium glycollate, cholinium propionate, cholinium lactate, choline chloride	288.15	[81]
PEG-600, PEG-1000, PEG-2000, PEG-3400	[C_8_NH_3_][OAc]	323.15	[84]
PEG-3500	[C_n_mim]Cl (n = 2~10)	298.15	[131]
PEG-1500	[C4mim]Cl	323.15, 333.15	[132]
PEG-4000, PEG-6000, PEG-8000,	[C_6_mim][C_12_SO_3_]	298.15	[133]
PEG-400, PPG-400	[Ch]Cl, [Ch][DHcit], [Ch][Bit], [Ch][Bic], [Ch][DHp], [Ch][Ac]	298.15	[137]
PPG-400	[Ch][BES]	298.15	[140]
PEG-6000	[C_2_mim][BF_4_], [C_4_mim][BF_4_], [C_2_mim]Br, [C_4_mim]Br	298.15	[141]
PEG-2000, PEG-4000, PEG-6000	[C_6_mim][C_12_SO_3_]	298.15	[142]

**Table 3:** Some surfactants/saccharides commonly used in the IL-based ATPSs.

Surfactants/Saccharides	ILs	Temperature/K	Refs.
Glucose	[C_3_mim][BF_4_], [C4mim][BF_4_]	242.15~308.15	[69]
Fructose	[C_4_mim][BF_4_]	298.15~318.15	[70]
Glucose, sucrose, maltose, and xylose	[C_n_mim][BF_4_] (n = 3~10)	242.15~373.15	[73]
SDBS	[C_4_mim][BF_4_]	283.15~303.15	[143]
SDS	[C_4_min]Cl, [C_6_mim]Cl, [C_8_mim]Cl	303.15	[145]
SDS, SDBS	[C_2_pi][BF_4_], [Phpi][BF_4_]	298.15~323.15	[134]
Triton X-100, Triton X-102	[C_2_mim][C_2_SO_4_], [Ch]Cl	298.15~333.15	[147,148]
Tween 20, Tween 80	[Ch]Cl, [N_1112OH_][C_4_H_5_O_6_]	293.2~333.15	[149,152]
Triton X-100	[C_2_mim]Cl	288.15~308.15	[156]
D-(+)-Glucose, D-(+)-galactose, D-(+)-fructose, D-(+)-mannose, D-(-)-arabinose, L-(+)-arabinose, D-(+)-xylose, D-(+)-maltose, D-sorbitol, maltitol, xylitol, sucrose	[C_2_C_1_py][C_4_F_9_SO_3_], [N_1112OH_][C_4_F_9_SO_3_], [C_2_mim][C_4_F_9_SO_3_], [C_2_mim][CF_3_SO_3_], [C_4_mim][CF_3_SO_3_], [C_2_C_1_pyr][CF_3_SO_3_]	298.15	[157]

## Data Availability

Not applicable.

## Abbreviation

[C_2_mim]Cl	1-Ethyl-3-methylimidazolium chloride
[C_4_mim]Cl	1-Butyl-3-methylimidazolium chloride
[C_5_mim]Cl	1-Pentyl-3-imidazolium chloride
[C_6_mim]Cl	1-Hexyl-3-methylimidazolium chloride
[C_7_mim]Cl	1-Heptyl-3-methylimidazolium chloride
[C_4_mmim]Cl	1-Butyl-2,3-dimethylimidazolium chloride
[C_4_py]Cl	*N*-Butylpyridinium chloride
[N_4 4 4 4_]Cl	Tetrabutylammonium chloride
[P_4 4 4 4_]Cl	Tetrabutylphosphonium chloride
[C_4_C_1_pyr]Cl	1-Butyl-1-methylpyrrolidinium chloride
[C_4_C_1_pip]Cl	1-Butyl-1-methylpiperidinium chloride
[C_4_C_1_py]Cl	1-Butyl-4-methylpyridinium chloride
[C_2_mim]Br	1-Ethyl-3-methylimidazolium bromide
[C_4_mim]Br	1-Butyl-3-methylimidazolium bromide
[C_6_mim]Br	1-Hexyl-3-methylimidazolium bromide
[C_8_mim]Br	1-Octyl-3-methylimidazolium bromide
[C_2_mim][CH_3_CO_2_]	1-Ethyl-3-methylimidazolium acetate
[C_2_mim][MeSO_4_]	1-Ethyl-3-methylimidazolium methylsulfate
[C_2_mim][C_2_SO_4_]	1-Ethyl-3-methylimidazolium ethylsulfate
[C_2_mim][CH_3_SO_3_]	1-Ethyl-3-methylimidazolium methanesulfonate
[C_2_mim][CF_3_SO_3_]	1-Ethyl-3-methylimidazolium trifluoromethanesulfonate
[C_4_mim][CH_3_CO_2_]	1-Butyl-3-methylimidazolium acetate
[C_4_mim][N(CN)_2_]	1-Butyl-3-methylimidazolium dicyanamide
[C_4_mim][CH_3_SO_3_]	1-Butyl-3-methylimidazolium methanesulfonate
[C_4_mim][HSO_4_]	1-Butyl-3-methylimidazolium hydrogensulfate
[C_4_mim][CF_3_SO_3_]	1-Butyl-3-methyl-imidazolium trifluoromethanesulfonate
[C_4_mim][TFA]	1-Butyl-3-methylimidazolium trifluoroacetate
[TBA][But]	Tetrabutylammonium butanoate
[TBA][Pent]	Tetrabutylammonium pentanoate
[TBA][Hex]	Tetrabutylammonium hexanoate
[TBA][Hept]	Tetrabutylammonium heptanoate
[TBA][Oct]	Tetrabutylammonium octanoate
[TBA][Dec]	Tetrabutylammonium decanoate
[C_4_mim][BF_4_]	1-Butyl-3-methylimidazolium tetrafluoroborate
[NH_4_][OAc]	Ammonium acetate
[C_3_NH_3_][OAc]	Propylammonium acetate
[C_4_NH_3_][OAc]	Butylammonium acetate
[C_6_NH_3_][OAc]	Hexylammonium acetate
[C_8_NH_3_][OAc]	Octylammonium acetate
[C_4_NH_3_][But]	Butylammonium butanoate
[Amim]Cl	1-Allyl-3-methylimidazolium chloride
[Ch]Cl	Cholinium chloride
[N_4 4 4 1_][NO_3_]	Tributylmethylammonium nitrate
[EOMiM]Br	1-(2-Methoxyethyl)-3-methylimidazolium bromide
[C_4_py]OTF	*N*-Butylpyridinium trifluoromethanesulfonate
[C_4_py][BF_4_]	*N*-Butylpyridinium tetrafluoroborate
[C_4_mim][NO_3_]	1-Butyl-3-methylimidazolium nitrate
[C_6_mim][NO_3_]	1-Hexyl-3-methylimidazolium nitrate
[C_2_mim]DCA	1-Ethyl-3-methylimidazolium dicyanamide
[C_3_mim]DCA	1-Propyl-3-methylimidazolium dicyanamide
[C_4_mim]DCA	1-Butyl-3-methylimidazolium dicyanamide
[C_6_mim]DCA	1-Hexyl-3-methylimidazolium dicyanamide
[C_4_C_1_pyr]DCA	1-Butyl-1-methylpyrrolidinium dicyanamide
[Ch][L-Pro]	Choline L-proline
[Ch][L-Cys]	Choline L-cysteine
[Ch][L-His]	Choline L-histidine
[Ch][L-Val]	Choline L-valine
[Ch][L-Ser]	Choline L-serine
[Ch][L-Met]	Choline L-methionine
[Ch][L-Ala]	Choline L-alanine
[C_2_Tr]Br	*N*-Ethyl-tropinium bromide
[C_3_Tr]Br	*N*-Propyl-tropinium bromide
[C_4_Tr]Br	*N*-Butyl-tropinium bromide
[C_5_Tr]Br	*N*-Pentyl-tropinium bromide
[C_2_Qn]Br	*N*-Ethyl-quinolinium bromide
[C_3_Qn]Br	*N*-Propyl-quinolinium bromide
[C_4_Qn]Br	*N*-Butyl-quinolinium bromide
[C_5_Qn]Br	*N*-Pentyl-quinolinium bromide
[C_6_Qn]Br	*N*-Hexyl-quinolinium bromide
[C_2_mim][OAc]	1-Ethyl-3-methylimidzaolium acetate
[C_4_MDEA]Br	*N*-methyl-*N*-butyl-*N,N*-di(hydroxyethyl)ammonium bromide
[C_6_MDEA]Br	*N*-methyl-*N*-hexyl-*N,N*-di(hydroxyethyl)ammonium bromide
[C_8_MDEA]Br	*N*-methyl-*N*-octyl-*N,N*-di(hydroxyethyl)ammonium bromide
[C_10_MDEA]Br	*N*-methyl-*N*-decyl-*N,N*-di(hydroxyethyl)ammonium bromide
[C_4_BDEA]Br	*N,N*-di(butyl)-*N,N*-di(hydroxyethyl)ammonium bromide
[P_4 4 4 4_][NO_3_]	Tetrabutylphosphonate nitrate
[N_4 4 4 4_][NO_3_]	Tetrabutylammonium nitrate
[Ch][Leu]	Choline leucine
[Ch][Gly]	Choline glycine
[Ch][Lys]	Choline lysine
[C_2_TMG][TEMPO-OSO_3_]	Ethyl-1,1,3,3-tetramethylguanidinium 4-sulfonatooxy-2,2,6,6-tetramethyl piperidine-1-yloxyl
[C_3_TMG][TEMPO-OSO_3_]	Propyl-1,1,3,3-tetramethylguanidinium 4-sulfonatooxy-2,2,6,6-tetramethyl piperidine-1-yloxyl
[C_4_TMG][TEMPO-OSO_3_]	Butyl-1,1,3,3-tetramethylguanidinium 4-sulfonatooxy-2,2,6,6-tetramethyl piperidine-1-yloxyl
[N_1 1 2 2OH_][TEMPO-OSO_3_]	Ethyl-(2-hydroxyethyl)-dimethylammonium 4-sulfonatooxy-2,2,6,6-tetramethyl piperidine-1-yloxyl
[N_1 1 3 2OH_][TEMPO-OSO_3_]	Propyl-(2-hydroxyethyl)-dimethylammonium 4-sulfonatooxy-2,2,6,6-tetramethyl piperidine-1-yloxyl
[N_1 1 4 2OH_][TEMPO-OSO_3_]	Butyl-(2-hydroxyethyl)-dimethylammonium 4-sulfonatooxy-2,2,6,6-tetramethyl piperidine-1-yloxyl
[N_1 1 5 2OH_][TEMPO-OSO_3_]	Pentyl-(2-hydroxyethyl)-dimethylammonium 4-sulfonatooxy-2,2,6,6-tetramethyl piperidine-1-yloxyl
[C_6_mim][C_12_SO_3_]	1-Hexyl-3-methyl imidazolium dodecyl sulfonate
[C_2_mim][SCN]	1-Ethyl-3-methylimidazolium thiocyanate
[C_4_mim][SCN]	1-Butyl-3-methylimidazolium thiocyanate
[Ch][L-Ala]	Cholinium L-alaninate
[Ch][DHcit]	Cholinium dihydrogen citrate
[Ch][Bit]	Cholinium bitartrate
[Ch][Bic]	Cholinium bicarbonate
[Ch][DHp]	Choline dihydrogen phosphate
[Ch][Ac]	Cholinium acetate
[Ch][BES]	Cholinium 2-[bis(2-hydroxyethyl)amino]ethanesulfonate
UCON	Poly(ethylene glycol-ran-propylene glycol) monobutyl ether
SDBS	Sodium dodecyl benzene sulfonate
SDS	Sodium dodecyl sulfonate
[C_2_pi][BF_4_]	1-Ethylpiperazinium tertrafluoroborate
[Phpi][BF_4_]	1-Phenylpiperazinium tertrafluoroborate
[N_1 1 1 2OH_][C_4_H_5_O_6_]	Cholinium bitartrate
[C_2_mim][BF_4_]	1-Ethyl-3-methylimidazolium tetrafluoroborate
[C_2_C_1_py][C_4_F_9_SO_3_]	1-Ethyl-3-methylpyridinium perfluorobutanesulfonate
[C_2_mim][C_4_F_9_SO_3_]	1-Ethyl-3-methylimidazolium perfluorobutanesulfonate
[N_1 1 1 2OH_][C_4_F_9_SO_3_]	Cholinium perfluorobutanesulfonate
[C_2_C_1_pyr][CF_3_SO_3_]	1-Ethyl-1-methylpyrrolidinium triflate
DIMCARB	*N,N*-dimethylammonium *N′,N′*-dimethylcarbamate
DPCARB	*N,N*-dipropylammonium *N′N′*-dipropylcarbamate
DACARB	*N,N*-diallylammonium *N′N′*-diallylcarbamate
DBCARB	Bis(2-ethylhexyl)ammonium bis(2-ethylhexyl)carbamate
